# Label-free wide-field imaging of brain tumors using near-infrared endogenous fluorescence and reflectance normalization

**DOI:** 10.1117/1.JBO.31.8.086005

**Published:** 2026-08-12

**Authors:** Gabriel Beaudoin, Victor Blanquez-Yeste, Frédérick Dallaire, Seung Soo Lee, Romain Cayrol, Marie-Christine Guiot, Mark A. Trifiro, Miltiadis Paliouras, Kevin Petrecca, Frédéric Leblond

**Affiliations:** a Polytechnique Montréal, Department of Physics Engineering, Montréal, Quebec, Canada; b Centre de recherche du Centre hospitalier de l’Université de Montréal (CRCHUM), Montréal, Quebec, Canada; c McGill University, Division of Clinical and Translational Research, Department of Medicine, Montreal, Quebec, Canada; d Jewish General Hospital, Lady Davis Institute for Medical Research, Montreal, Quebec, Canada; e Department of Pathology, Centre Hospitalier de l’Université de Montréal (CHUM), Montréal, Quebec, Canada; f McGill University, Department of Pathology, Montreal, Quebec, Canada; g McGill University, Montreal Neurological Institute-Hospital, Montreal, Quebec, Canada; h Jewish General Hospital, Division of Endocrinology, Montreal, Quebec, Canada

**Keywords:** endogenous fluorescence, near-infrared imaging, wide-field imaging, label-free contrast, spectroscopy, neurosurgery, astrocytoma, glioblastoma, intraoperative guidance.

## Abstract

**Significance:**

Maximizing safe tumor resection remains a major challenge in brain tumor surgery due to the lack of reliable real-time intraoperative contrast between tumor and healthy brain tissue. Label-free optical methods based on endogenous tissue fluorescence could provide simple, noninvasive feedback to guide resection while avoiding the logistical and regulatory limitations of exogenous fluorophores.

**Aim:**

The study aims to investigate whether endogenous near-infrared (NIR) fluorescence can differentiate tumor from normal brain tissue *in vivo* and to develop a wide-field fluorescence and reflectance imaging system for label-free visualization of brain tumor specimens *ex vivo*.

**Approach:**

We analyzed *in vivo* data acquired with a hand-held near-infrared spectroscopy probe from 26 patients (430 measurements) to quantify endogenous fluorescence differences between glioblastoma or astrocytoma and normal brain. Based on these findings, we built a wide-field imaging prototype using 808-nm laser excitation, red-shifted fluorescence detection with an InGaAs short-wave infrared camera, and co-registered reflectance imaging for ratiometric normalization. The system was validated with *ex vivo* human surgical specimens (14 samples from five patients).

**Results:**

Endogenous fluorescence intensity measured *in vivo* was significantly higher in normal brain tissue compared with tumor, yielding positive predictive values of 85 to 88% for tumor and a negative predictive value of 96% for normal tissue. *Ex vivo* wide-field imaging reproduced this contrast: normalized fluorescence was lower in tumor regions than in histologically confirmed normal areas, consistent with metabolic differences observed *in vivo*.

**Conclusions:**

Endogenous NIR fluorescence provides intrinsic contrast between normal and tumoral brain tissue. The demonstrated corrected wide-field fluorescence imaging approach could offer a feasible, label-free method for real-time visualization of tumor margins, supporting its future clinical translation as an intraoperative guidance tool.

## 1 Introduction

Surgical resection remains a cornerstone of treatment for brain tumors, including glioblastoma and astrocytoma.^1^^–^^3^ Achieving maximal safe resection is one of the most important prognostic factors for patient survival and recurrence-free outcome.^4^^,^^5^ However, complete tumor removal is often limited by the difficulty of differentiating tumor margins from normal brain tissue using standard white-light microscopy.^6^ For navigation, neurosurgeons must rely on preoperative magnetic resonance imaging (MRI), but these maps do not update intraoperatively and are impacted by brain shift during surgery, which results in loss of spatial accuracy.^7^^,^^8^ Consequently, resection decisions are guided primarily by visual and tactile cues, balancing oncologic completeness with the risk of postoperative neurological deficits involving motor, visual, or cognitive functions.^9^ Real-time intraoperative imaging tools that provide objective, label-free, and spatially resolved contrast between tumor and normal tissue could therefore greatly improve surgical precision and patient outcomes.

A wide-field optical imaging method is particularly appealing in this context. Wide-field systems can provide real-time spatial maps over the surgical field, maintaining anatomical context and supporting continuous surgical workflow. The ability to rapidly visualize large surface areas without contact would allow for effective intraoperative assessment of tissue boundaries and complement existing guidance modalities. Endogenous near-infrared (NIR) imaging could provide benefits in that respect. The NIR spectral window (700 to 1000 nm) minimizes absorption by hemoglobin and water, reduces scattering, and enables deeper light penetration into tissue.^8^ However, endogenous NIR fluorescence remains relatively underexplored due to the technical challenges of detecting weak signals at these wavelengths. Combining autofluorescence with reflectance imaging could also provide an opportunity to improve and normalize this intrinsic contrast.^10^ Reflectance images acquired at the excitation wavelength can correct spatial variations in illumination, tissue topography, and optical attenuation, giving a ratiometric fluorescence-to-reflectance signal that isolates biochemical differences from purely structural or geometric effects.^11^

Endogenous tissue fluorescence offers a promising, label-free source of contrast. Autofluorescence arises primarily from endogenous fluorophores such as reduced nicotinamide adenine dinucleotide (NADH), flavins (FAD), porphyrins, lipopigments, and structural proteins such as collagen.^12^ Alterations in their relative concentrations and oxidation states coming from malignant transformation reflect changes in cellular metabolism and tissue composition.^13^ Exogenous fluorophores have been used to demonstrate the clinical utility of fluorescence guidance in oncology, especially in neurosurgery.^14^ Five-aminolevulinic acid (5-ALA), which induces accumulation of protoporphyrin IX (PpIX) in tumor cells, is frequently used in fluorescence-guided glioblastoma surgery and has improved resection rates and survival outcomes.^15^ Other agents such as methylene blue and indocyanine green (ICG) have been explored for a variety of surgical applications,^15^ with ICG offering strong NIR emission and favorable biocompatibility. However, the adoption of all labeled approaches can be limited because they require preoperative preparation, regulatory approval, and patient-specific dosing. Label-free methods based on endogenous contrast would therefore provide a simpler and safer alternative for intraoperative use.

In this study, we present a two-part investigation of label-free NIR fluorescence imaging for brain tumor discrimination. First, we analyze *in vivo* endogenous fluorescence data collected from 26 patients using a spectroscopy probe optimized for inelastic scattering detection, comprising 430 measurements in glioblastoma and astrocytoma.^16^^,^^17^ These results demonstrate that endogenous fluorescence can effectively differentiate tumor from normal brain, with positive predictive values of 85 to 88% for tumor and a negative predictive value of 96% for normal brain. Fluorescence intensity was consistently higher in normal tissue, suggesting differences in metabolic profiles between healthy and neoplastic regions.

Building on these findings, we developed a custom wide-field NIR fluorescence and reflectance imaging system designed for *ex vivo* evaluation of brain tumor specimens. The system uses 808 nm laser excitation with expanded-beam illumination to capture red-shifted emission with an InGaAs short-wave infrared (SWIR) camera (also sensitive in the NIR, with a spectral range from 500 to 1700 nm) through a high-pass filter. Corresponding reflectance images at the excitation wavelength were used to make ratiometric normalization of fluorescence signals possible and compensate for spatial illumination and surface variations. We report imaging results from 14 specimens obtained from five patients with glioblastoma and astrocytoma, along with corresponding histological analyses. Significant differences in normalized fluorescence between tumor and normal regions were observed, consistent with *in vivo* probe data, although with smaller contrast magnitude, likely reflecting post-resection metabolic changes.

Together, these studies demonstrate that endogenous NIR fluorescence provides contrast between tumor and normal brain tissue, both *in vivo* and *ex vivo*, and that wide-field imaging of this signal is feasible using simple hardware. This work establishes a foundation for future intraoperative translation of wide-field endogenous fluorescence imaging as a complementary tool for real-time surgical guidance.

## 2 Materials and Methods

### 2.1 *In Vivo* Optical Spectroscopy Data Acquisition and Analysis

We reanalyzed a subset of *in vivo* spectroscopy data acquired during a previously published clinical study on intraoperative brain tumor detection at the Montreal Neurological Institute–Hospital (MNI-H, Montreal, Canada).^16^^,^^17^ The study was a prospective observational trial evaluating the diagnostic performance of inelastic scattering signals (Raman spectroscopy) during open-cranium tumor resections. All patients provided informed consent, and all procedures adhered to institutional ethical guidelines.

The previous study focused on a subset of 26 patients, either with glioblastoma (n=24) or astrocytoma (n=2). Data from other patients with either meningioma or metastases to the brain were excluded, as no *ex vivo* specimen imaging was performed for these conditions in the current study. The dataset contained 430 Raman spectra collected intraoperatively from histologically confirmed sites of glioblastoma, astrocytoma, and normal brain. In total, 287 measurements were made in glioblastoma bulk tumor, 18 in astrocytoma bulk tumor, and 125 in normal brain. The Raman system (Sentry Research Unit; Reveal Surgical) consisted of a sterilizable handheld probe illuminated by a 785-nm continuous-wave laser. The device collected scattered light through nine surrounding optical fibers coupled to a compact spectrometer equipped with a thermoelectrically cooled CCD. Each spectrum spanned 400 to $2000\;{\mathrm{cm}}^{-1}$ at $∼1.8\;{\mathrm{cm}}^{-1}$ resolution. Regions of interest for measurements were defined using preoperative MRI: contrast-enhancing T1-weighted regions corresponding to bulk tumor and non-enhancing brain areas representing normal brain.

Spectral preprocessing followed the standardized pipeline reported in our previous work.^18^ Briefly, each acquisition underwent dark-count subtraction, cosmic-ray removal, wavenumber calibration, instrument-response correction, and averaging of repeated spectra. A fluorescence baseline was extracted using the BubbleFill algorithm ($\text{minimum bubble parameter}=50\;{\mathrm{cm}}^{-1}$) and stored separately prior to subtraction of the Raman-specific component. In the present analysis, this extracted baseline, corresponding to the broad unstructured background of each spectrum, was interpreted as endogenous *in vivo* fluorescence.

For each measurement, the mean fluorescence intensity was computed across the baseline-fitted curve, providing a quantitative parameter representing endogenous NIR emission following 785 nm tissue excitation. Spectra were grouped into three categories: glioblastoma (bulk tumor), astrocytoma (bulk tumor), and normal brain. Data were pooled across patients within each category. Boxplots were generated to visualize fluorescence intensity distributions, and MannWhitney U testing was applied to assess differences between tumor and normal tissue for each tumor type independently. Positive and negative predictive values were derived using histopathology as the reference standard, based on a receiver operating characteristic analysis.

This analysis provided the foundation for the current imaging study by confirming that endogenous fluorescence, derived directly from Raman background intensity, differentiates tumor from normal brain with high sensitivity and specificity. These quantitative *in vivo* results guided the design and optimization of the wide-field NIR fluorescence imaging system described in the following sections.

### 2.2 Imaging System Design

A custom multimodal imaging platform was developed to acquire wide-field images of biological tissues in the NIR range, supporting two modes of operation: fluorescence and reflectance imaging. The system was designed to provide flexible spatial coverage, minimize optical losses and artifacts, and allow modular integration of additional illumination wavelengths within the NIR spectrum. Mechanical distances between components were optimized to facilitate rapid changes in the field of view while leaving sufficient space for interchangeable filters, neutral density elements, and auxiliary illumination modules.

The excitation source consisted of an 808-nm LED laser (*Arktis Laser*, LRD-0808 Series, 5000 mW maximum output) coupled to a 3-mm-core liquid light guide (LLG3-4Z). The diverging output from the light guide was collimated using a focusing lens and directed through a linear polarizer (Thorlabs LPNIRE100-B, N-BK7 windows). The beam was then transmitted through a polarizing dichroic mirror (Thorlabs, PBS255, Newton, New Jersey, United States), resulting in an epi-illumination setup compatible with single or multiple NIR excitation wavelengths. This geometry minimized specular reflections from the tissue surface and stopped back-reflected light from reaching the detector.^19^ The use of crossed polarizers further suppressed surface glare and ensured well-defined relative polarization of the incident and detected light.

On the detection side, a pair of Thorlabs NIR achromatic doublets provided 5× magnification, corresponding to a field of view of ∼7×8  mm. Rapid transitions between fluorescence and reflectance modes were made possible by two motorized filter wheels. 1) Reflectance mode: diffusely reflected illumination at the excitation wavelength was captured using an 800-nm long-pass filter (Thorlabs, FELH0800). A secondary polarizer was positioned orthogonally to the illumination polarization to reject specular reflection and suppress internal optical interference. 2) Fluorescence mode: an 850-nm long-pass filter (Thorlabs, FELH0850) collected red-shifted emission from endogenous or exogenous fluorophores, while two notch filters (Thorlabs, 808 nm center wavelength, 34-nm half-width, optical density 5.5) blocked residual excitation light and further attenuated specular reflections. Images were acquired using a Photon, etc., Alizé 1.7v InGaAs camera cooled to −50°C to minimize dark noise.

Table 1 shows the clinical and pathological characteristics of the patient cohort included in this study. The table summarizes patient identifiers, histopathological diagnosis (glioblastoma or astrocytoma), the number of *ex vivo* specimens obtained per patient, and the predominant tissue types identified within each specimen based on histopathological assessment. Tissue categories include tumor, normal brain, necrotic regions, and blood contamination.

**Table 1:** Summary of patient data, diagnostics, and pathologist predictions.

Patient ID	Diagnosis	#Samples	Pathologist prediction	Included
P2	Meningioma	2	Necrosis, normal and tumor tissue	No
P3	Meningioma	2	Necrosis and tumor tissue	No
P6	Gliobatoma	2	Tumor tissue	No
P7	Gliobastoma	2	Normal and tumor tissue	Yes
P8	Gliobatoma	2	Necrosis and tumor tissue	Yes
P11	Astrocytoma	2	Blood and tumor tissue	Yes
P12	Astrocytoma	3	Normal tissue	Yes
P14	Gliobastoma	5	Normal and tumor tissue	Yes

### 2.3 Patient Inclusion and *Ex Vivo* Brain Tissue Samples

Patients were recruited from an ongoing *in vivo* brain tumor spectroscopy study approved by the institutional ethics committee. Candidates for *ex vivo* imaging were selected intraoperatively by the neurosurgeon based on tumor size and accessibility for safe tissue sampling. Among the first 14 enrolled patients, nine provided *ex vivo* samples. All underwent open-cranium resections for histologically confirmed brain tumors, either glioblastoma or astrocytoma. The first three *ex vivo* specimens were excluded from analysis due to excessive specular reflections observed in early imaging trials. Subsequent system modifications eliminated this issue. Table 1 summarizes the clinical and pathological characteristics of all patients who provided *ex vivo* tissue.

Following resection, fresh tissue specimens were collected, placed in phosphate-buffered saline (PBS) to avoid dehydration, and immediately transferred to the imaging station. Each specimen (typically 1 to 2 cm in size) was bisected along its longitudinal axis to produce two flat surfaces suitable for imaging. Minimal PBS coverage was maintained to prevent desiccation while avoiding excess liquid that could increase surface scattering and glare. After imaging, the sides of each specimen were inked to preserve orientation for histopathological correlation. The ink was fixed using mordant before formalin fixation. Samples were fixed in 10% formalin for up to 24 h prior to paraffin embedding and subsequent histological processing.

### 2.4 Label-Free Imaging Procedures

Once the InGaAs detector reached its operating temperature of −50°C, samples were put under ambient white-light illumination for orientation. First, imaging was done in fluorescence mode. The filter wheels were placed in the fluorescence configuration (an 850 nm long-pass filter plus two 808 nm notch filters), and the excitation source was set to give 200 mW at the imaging plane for $176\;\mathrm{mW}/{\mathrm{cm}}^{2}$. Fluorescence images were taken with exposure times of 30, 60, 150, and 300 s, each with up to four accumulations to improve signal-to-noise ratio.

Subsequently, the system was switched to reflectance mode (800 nm long-pass filter only), and a single reflectance image was acquired with 1 ms exposure. Immediately afterward, a dark image was captured with the laser turned off and the same exposure time as the reflectance acquisition to record camera noise. The entire imaging sequence was performed in a fixed order to minimize sample motion between acquisitions and reduce the total time before fixing the specimen. All images, except the initial white-light photographs used for orientation, were stored, containing pixel intensity data (640×512  pixels×1 accumulation) and acquisition metadata, including exposure time.

### 2.5 Data Processing and Statistical Analysis

Raw images were processed using a standardized pipeline. Both fluorescence and reflectance images were smoothed using median and Gaussian filters to suppress high-frequency noise. Fluorescence images were then normalized by reflectance intensity on a pixel-wise basis to produce ratiometric fluorescence maps that compensate for surface geometry and illumination variability. A binary mask was generated for each specimen to exclude pixels corresponding to nontissue regions or low-signal areas near image edges.

Histopathological annotations were used to segment each image into tumor, necrotic, and normal regions. Areas associated with blood vessels were also identified. Corresponding masks were registered to the imaging data to extract mean fluorescence and reflectance intensities per tissue class. Normality of the data was assessed separately for each pathology using the ShapiroWilk test on the distributions of mean fluorescence and reflectance values across samples. Due to the limited number of samples, normality could not be reliably assessed for blood and necrosis, and these tissue types were therefore excluded from parametric statistical analyses. Statistical analysis was performed using two parametric approaches: (1) a paired t-test comparing tumor versus normal regions within each specimen and (2) mixed-effects ANOVA models with tissue type as a fixed effect and specimen as a random effect to account for inter-patient variability. In an attempt to include results from necrosis, nonparametric tests were also considered, but these are limited by small sample size and nonindependence of observations. All statistical computations were performed using Python (SciPy, StatsModels).

## 3 Results

### 3.1 Endogenous *In Vivo* Fluorescence Derived from Raman Spectra

**Fig. 1:**
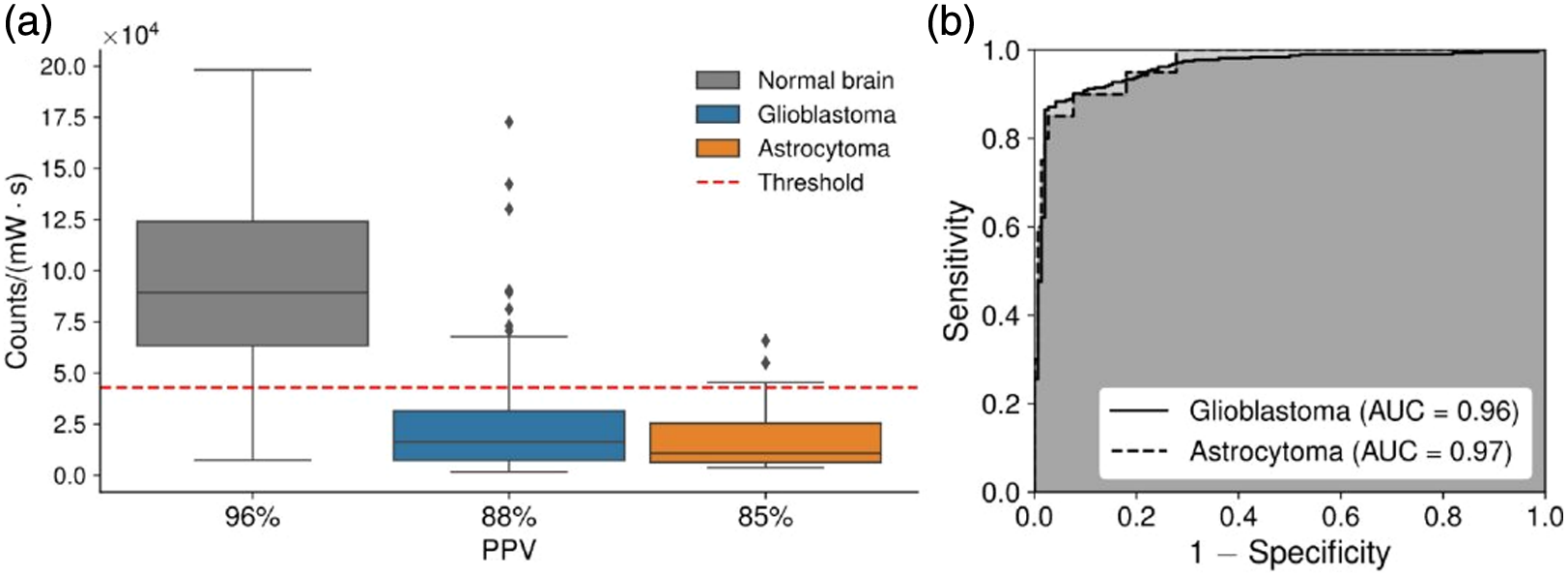
Quantitative analysis of endogenous *in vivo* fluorescence extracted from the baseline (background) of Raman spectra acquired intraoperatively with a handheld Raman probe. (a) Boxplots show fluorescence intensity distributions for three tissue categories: bulk glioblastoma, bulk astrocytoma, and normal brain. The fluorescence intensity corresponds to the broad background signal underlying the Raman spectrum, reflecting endogenous tissue emission under 785 nm excitation. Univariate statistical analysis revealed statistically significant differences (p<0.001) between normal brain and both tumor classes, with fluorescence consistently higher in normal tissue. (b) Receiver operating characteristic (ROC) curve obtained by grouping all tumor spectra versus normal brain, using endogenous fluorescence intensity as the classifier variable. The optimal operating point (upper-left corner of the ROC curve) yielded a positive predictive value (PPV) of 85 to 88% for tumor detection and a negative predictive value (NPV) of 96% for normal brain, confirming the discriminative power of endogenous fluorescence as a label-free biomarker *in vivo*.

Endogenous fluorescence extracted from the baseline of intraoperative Raman spectra demonstrated clear differences between tumor and normal brain tissue. Figure 1(a) presents boxplots of the background fluorescence intensity for bulk glioblastoma, bulk astrocytoma, and normal brain, showing consistently higher fluorescence in normal tissue than in both tumor types (p<0.001). A receiver operating characteristic (ROC) analysis performed by grouping all tumor spectra against normal brain [Fig. 1(b)] yielded a positive predictive value (PPV) of 85 to 88% for tumor and a negative predictive value (NPV) of 96% for normal tissue. These results confirm that the broadband fluorescence underlying the Raman signal could serve as a reliable, label-free optical biomarker that distinguishes malignant from normal brain tissue *in vivo*.

### 3.2 *Ex Vivo* Imaging of Human Brain Tumor Specimens

**Fig. 2:**
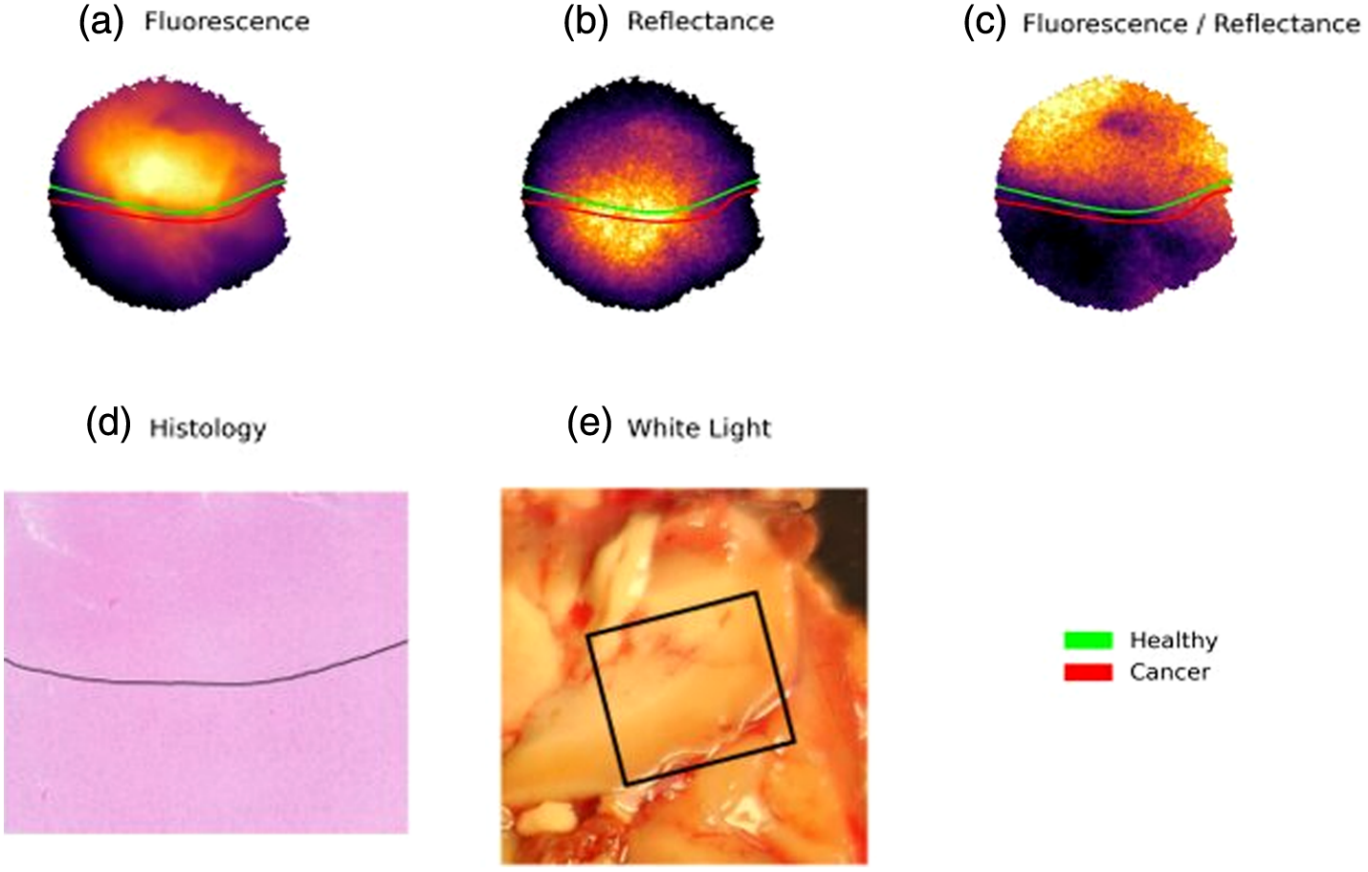
Representative *ex vivo* images were acquired from specimen 3 of patient 14 (glioblastoma) using the wide-field near-infrared fluorescence and reflectance imaging system. Panels show (a) raw fluorescence image acquired under 808 nm excitation through the 850 nm long-pass filter; (b) corresponding diffuse reflectance image at 808 nm; (c) ratiometric fluorescence map obtained by dividing the fluorescence image by the normalized reflectance image, isolating intrinsic fluorescence contrast; (d) corresponding hematoxylin and eosin (H&E) histology section showing the spatial distribution of tumor and normal brain regions; and (e) gross photograph of the specimen surface after imaging. The ratiometric fluorescence image highlights reduced endogenous fluorescence intensity in tumor regions relative to surrounding normal tissue, consistent with the *in vivo* findings.

Figure 2 illustrates representative *ex vivo* images from specimen 3 of patient 14 (glioblastoma). The raw fluorescence image [Fig. 2(a)] reveals regional contrast corresponding to histologically distinct zones. Diffuse reflectance at 808 nm [Fig. 2(b)] captures spatial variations in illumination and surface geometry, particularly near edges. The ratiometric fluorescence-to-reflectance image [Fig. 2(c)] highlights reduced fluorescence intensity in tumor regions relative to surrounding normal tissue. Comparison with the gross photograph [Fig. 2(e)] and the corresponding H&E histology [Fig. 2(d)] confirms spatial alignment between areas of diminished fluorescence and tumor infiltration identified by pathology. These qualitative findings are consistent with the *in vivo* Raman results, reinforcing that endogenous fluorescence decreases in malignant tissue.

**Fig. 3:**
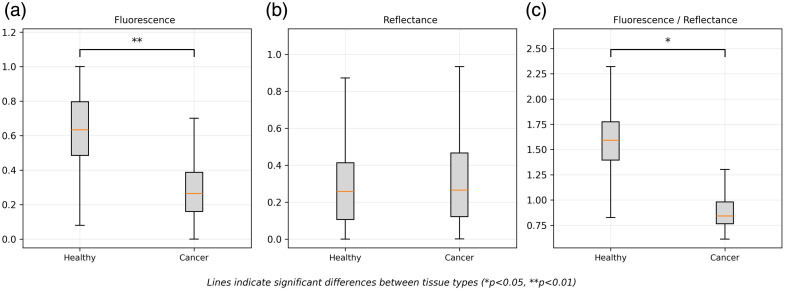
Quantitative analysis of optical signals from specimen 3 of patient 14 (glioblastoma) corresponding to the images shown in Fig. 1. Boxplots display the distribution, median, interquartile range, and outliers for regions histologically classified as tumor and normal brain. Panels show (a) raw fluorescence intensity, (b) raw reflectance intensity, and (c) fluorescence normalized by reflectance (F/R). p-values from paired t-tests are indicated on each plot, demonstrating statistically significant differences between tumor and normal tissue across all normalization methods (p<0.05).

Boxplots summarizing pixel intensity distributions for tumor and normal regions from the same specimen are shown in Fig. 3. Although reflectance correction slightly reduces absolute contrast, it improves signal uniformity and mitigates the influence of uneven illumination or surface curvature. The two main imaging parameters, raw fluorescence and fluorescence/reflectance, show statistically significant differences between tumor and normal tissue across samples containing both tissue types in the field of view (paired t-test, p<0.01 for raw fluorescence and p<0.05 for reflectance-corrected fluorescence). Paired t-tests were performed by comparing the mean intensity of each tumor–normal pair within a specimen. Nonparametric tests (Wilcoxon) were also considered to illustrate the direction of the effect (W=0, tumor<normal), but the test is limited by the small sample size (n=5), which constrains the minimum achievable p-value. These results demonstrate that both raw and normalized fluorescence metrics reliably distinguish tumor from normal brain within a single specimen.

### 3.3 Evaluation of Normalization Strategy in Normal Brain Tissue

**Fig. 4:**
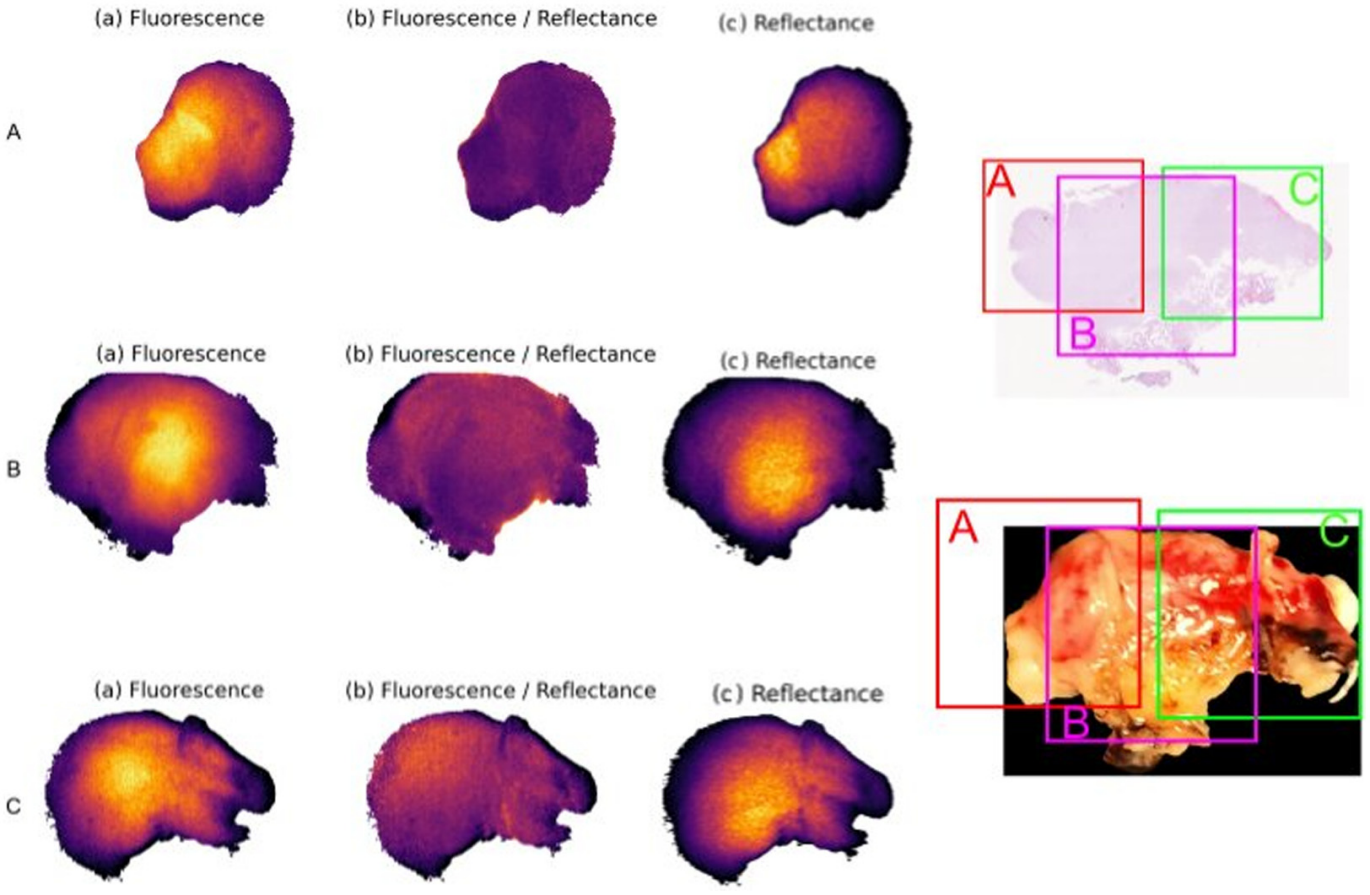
Demonstration of illumination and geometry normalization using fluorescence-to-reflectance ratios in a specimen histologically confirmed as normal brain tissue. The right panels show the gross photograph and corresponding hematoxylin and eosin (H&E) histology image, with colored squares indicating the locations of three regions of interest (ROIs) (A)–(C). For each ROI, five image modalities are displayed: (a) raw fluorescence; (b) fluorescence normalized by reflectance; and (c) raw reflectance. Rows correspond to ROI A (top), ROI B (middle), and ROI C (bottom). Despite visible heterogeneities in the raw fluorescence and reflectance signals caused by surface height variations and optical property differences, the fluorescence-to-reflectance normalization yields a spatially homogeneous signal across all regions, demonstrating the effectiveness of ratiometric correction in compensating for illumination nonuniformity and surface geometry effects.

To assess the normalization procedure in samples without pathology-related contrast, we analyzed specimens classified entirely as normal brain (Fig. 4). Three regions of interest (ROIs A-C) were selected from different areas of a single specimen. Raw fluorescence and reflectance images exhibited modest spatial variations caused by illumination profile and sample curvature. Normalization using reflectance substantially reduced these gradients, resulting in homogeneous fluorescence-to-reflectance maps across all ROIs. This confirms that the ratiometric correction effectively compensates for spatial heterogeneities in excitation and collection geometry, providing a more uniform signal baseline for comparison across specimens.

#### 3.3.1 Aggregate statistical analysis across all ex vivo specimens

**Fig. 5:**
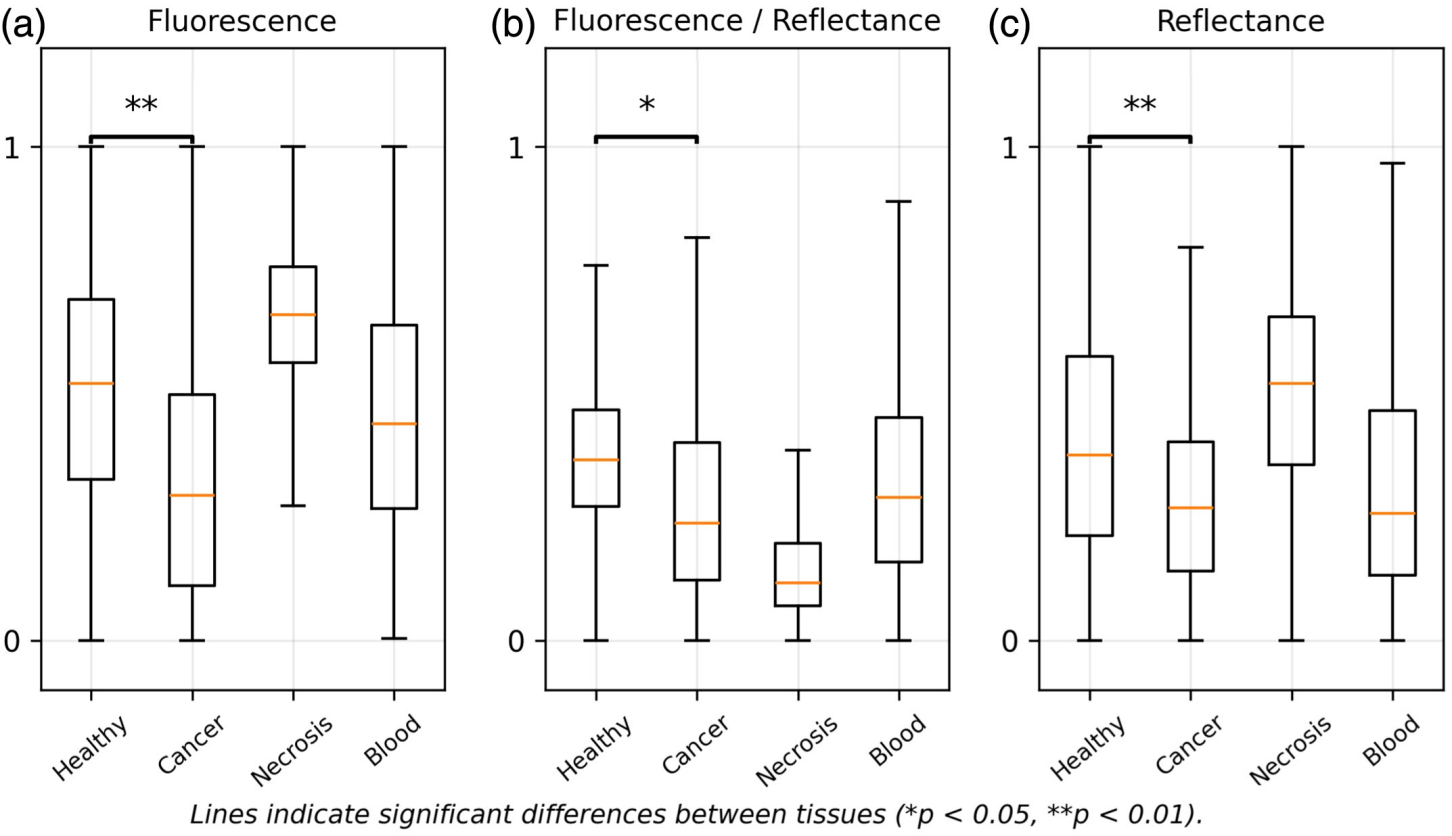
Quantitative analysis of *ex vivo* endogenous fluorescence and reflectance signals across all imaged specimens, grouped by histopathological classification. All pixels from the dataset were pooled and categorized as normal brain, tumor (glioblastoma and astrocytoma combined), necrosis, or blood, based on the neuropathologist’s assessment (see Table 1). Boxplots display the distribution, median, interquartile range, and outliers for each tissue class across five imaging-derived parameters: (a) raw fluorescence intensity, (b) fluorescence normalized by reflectance, and (c) raw reflectance intensity. p-values from mixed-effects ANOVA tests are indicated on each plot, demonstrating statistically significant differences (p<0.05) between normal brain and tumor tissue across all modalities.

To illustrate the imaging performance of the system, Figure 2 presents a representative example of *ex vivo* imaging results obtained from one glioblastoma specimen, showing fluorescence, reflectance, and normalized maps alongside the corresponding histology. For clarity and space considerations, representative images for all imaged specimens are provided in Fig. S1 in the Supplementary Material. The specimen shown in Fig. 2 was selected because it best demonstrates the expected contrast trends between normal brain and bulk tumor regions, consistent with the overall dataset. In other specimens, the visual distinction between tissue types was sometimes less pronounced. Nevertheless, when aggregating data from all patients and specimens (Fig. 5), statistically significant differences were observed between normal brain and tumor, confirming that the trends illustrated in the representative example are consistent across the full dataset.

To assess global trends, all imaged pixels from all patients were pooled and classified according to the neuropathologists’ annotations in Table 1 as normal brain, tumor, necrosis, or blood. Boxplots for each imaging parameter are shown in Fig. 5. Normal brain consistently exhibited higher fluorescence intensity than tumor regions across all modalities (p<0.05, mixed-effects ANOVA). Normalization using reflectance reduced inter-sample variability while preserving statistically significant differences between tissue classes. Necrotic regions displayed an initial high signal in both reflectance and fluorescence images, which could correlate with lower tissue absorption. After reflectance correction, the tendency reversed, and necrosis showed the lowest signal. Blood-containing regions displayed the lowest reflectance, consistent with strong absorption at 808 nm. These findings corroborate the *in vivo* observations and confirm that endogenous NIR fluorescence provides intrinsic contrast between healthy and pathological brain tissue *ex vivo*.

## 4 Discussion

This study investigated whether NIR endogenous fluorescence can serve as a label-free optical marker to distinguish tumor from normal brain tissue, using both *in vivo* point-based spectroscopy measurements and *ex vivo* wide-field imaging. Together, these results demonstrate that endogenous fluorescence, whether extracted from the broadband background of Raman spectra or captured directly through imaging, provides consistent contrast between tumor and normal brain. The findings establish a foundation for future development of real-time intraoperative imaging tools.

*In vivo* Raman measurements revealed that the broadband fluorescence background underlying the Raman signal was consistently higher in normal brain than in tumor for both glioblastoma and astrocytoma. The resulting positive and negative predictive values (85 to 88% and 96%, respectively) suggest that endogenous fluorescence may serve as a sensitive marker for tumor detection. However, because these single-point measurements were obtained from bulk tumor regions corresponding to T1 contrast-enhancing zones, no conclusion can be drawn regarding the invasive margins, where previous Raman spectroscopy studies demonstrated sensitivity to molecular infiltration signatures.^20^^,^^21^ Instead, the present fluorescence data suggest that endogenous emission may act as a bulk tumor marker, analogous in spatial specificity to contrast-enhancing MRI.

The *in vivo* Raman-based measurements were performed in contact mode, which eliminates certain optical artifacts, such as illumination inhomogeneity and surface curvature, that affect imaging systems. However, these single-point fluorescence intensities were not normalized for absorption or scattering. As a result, the measured fluorescence represents a compound index influenced by both intrinsic fluorophore concentration and tissue optical attenuation. The consistency across multiple patients, despite this limitation, indicates that even this mixed signal reflects the structural and metabolic differences between normal and malignant tissue and contains diagnostically significant information.

Unlike contact Raman measurements, wide-field fluorescence imaging is highly sensitive to illumination and geometric heterogeneities. To address this, the present imaging system implemented a reflectance-based normalization strategy. Our approach is based on the methodology proposed in previous work by Valdés et al., where reflectance-based normalization is used to bring fluorescence measurements closer to a corrected representation of tissue response. The goal of this method is not to retrieve absolute fluorescence intensity values but rather to obtain a normalized metric that better reflects relative signal variations across tissue regions. By dividing the fluorescence image by the co-registered reflectance image acquired at the excitation wavelength, the approach at least partially compensates for variations in excitation intensity, sample curvature, and local optical properties. The corrected ratios are computed prior to any subtraction-based processing steps (such as background subtraction or normalization procedures involving subtraction), which can affect both the numerator and denominator differently, potentially leading to over- or under-correction. In contrast, multiplicative factors under identical acquisition parameters only rescale the ratio image globally, without altering its spatial structure or relative contrast. The results demonstrated that normalization by reflectance produced spatially homogeneous fluorescence maps for tissue regions with uniform composition. The fluorescence-to-reflectance ratio produced more interpretable contrast than raw fluorescence. This finding highlights the importance of considering both illumination geometry and intrinsic tissue optics when quantifying fluorescence. Residual edge effects remained in samples smaller than the field of view or with pronounced curvature, reflecting limitations in the optical depth of field. Nonetheless, the ratiometric correction significantly reduced variability and improved correspondence between fluorescence maps and histologically defined regions.

Differences in physiological state between *in vivo* and *ex vivo* tissue are expected to influence fluorescence contrast. Following surgical resection, loss of perfusion and oxygenation alters hemoglobin absorption and scattering,^22^ while reduced metabolic activity might slightly lower levels of endogenous fluorophores.^23^ These changes likely contribute, in part, to the reduced absolute fluorescence and smaller tumor-to-normal contrast observed *ex vivo* compared with *in vivo* measurements. Despite these shifts, the directionality of contrast remained consistent: normal brain exhibited higher fluorescence than tumor in both datasets. This stability suggests that at least part of the observed fluorescence contrast arises from fundamental biochemical and metabolic differences, not from transient hemodynamic effects alone.

The mechanisms underlying endogenous NIR fluorescence contrast between tumor and normal brain likely reflect differences in tissue composition and metabolic state. The NIR region lies closer to the extended emission range of porphyrins and lipopigments than to that of NADH and FAD or structural proteins such as collagen, although their contribution to fluorescence variations likely reflects differences primarily known to emit in the visible range, they are included here as potential contributors to explain the fluorescence signal detected in previous Raman studies and in this article. In normal brain cells, higher oxidative metabolism and intact mitochondrial function may indirectly influence fluorescence through the abundance of certain fluorophores, potentially contributing to stronger NIR emission. In contrast, malignant regions, particularly glioblastomas, exhibit necrosis and hypoxia, which reduce NIR fluorescence primarily through loss of intact fluorophores, tissue structural changes, and increased absorption by blood.^24^^,^^25^ Metabolic reprogramming (Warburg effect) alters NADH and FAD levels, which contribute less to NIR autofluorescence than porphyrins and lipopigments, though they may still exert minor effects on this spectral region.^25^ The lower fluorescence observed in necrotic and blood-contaminated regions is therefore consistent with structural and optical attenuation. Thus, the reduced fluorescence signal in tumors likely reflects a combination of altered fluorophore content, tissue structure, and optical properties, which align with the histopathological characteristics of malignant tissue.

This work has several limitations. The *ex vivo* study included a limited number of patient specimens and tumor types, and no direct intraoperative wide-field measurements were performed. The delay between resection and imaging may have introduced metabolic and optical changes. The *in vivo* fluorescence data derived from Raman spectra were not normalized for scattering or absorption, preventing quantitative cross-comparison with imaging results. Nevertheless, the overall consistency between the *in vivo* and *ex vivo* datasets demonstrates the robustness of the fluorescence contrast. Future research will concentrate on implementing real-time reflectance normalization within the Raman probe and adapting the wide-field system to intraoperative imaging in living tissue. Correlation with metabolic and perfusion MRI, as well as with exogenous fluorescence-guided surgery techniques (e.g., 5-ALA), could further clarify the biological mechanisms of the observed contrast. Larger patient cohorts and inclusion of infiltrative tumor margins will be essential to assess sensitivity to invasion and to optimize the technology for clinical translation.

## 5 Conclusion

Endogenous near-infrared fluorescence measured either from point-based Raman spectra or through wide-field imaging could provide robust, label-free contrast between normal and tumor brain tissue. Although *in vivo* fluorescence primarily reflects a compound optical index influenced by tissue attenuation, reflectance-normalized fluorescence imaging gives a reliable, spatially uniform metric sensitive to intrinsic biochemical differences. These findings indicate that endogenous NIR fluorescence could serve as a bulk tumor marker, complementing contrast-enhanced MRI and potentially guiding real-time surgical decision-making without the need for exogenous agents.

## Supplementary Material

10.1117/1.JBO.31.8.086005.s01

## Data Availability

The data and materials information that support the findings of this study are available from the corresponding author upon reasonable request.
