# Supplementary material for: Label-free wide-field imaging of brain tumors using near-infrared endogenous fluorescence and reflectance normalization
Source: J Biomed Opt. 2026 Aug 12;31(8):086005. doi: 10.1117/1.JBO.31.8.086005 (PMC13463984; doi:10.1117/1.JBO.31.8.086005)
Supplement: Supplementary file 1 [file JBO_031_086005_SD001.pdf]

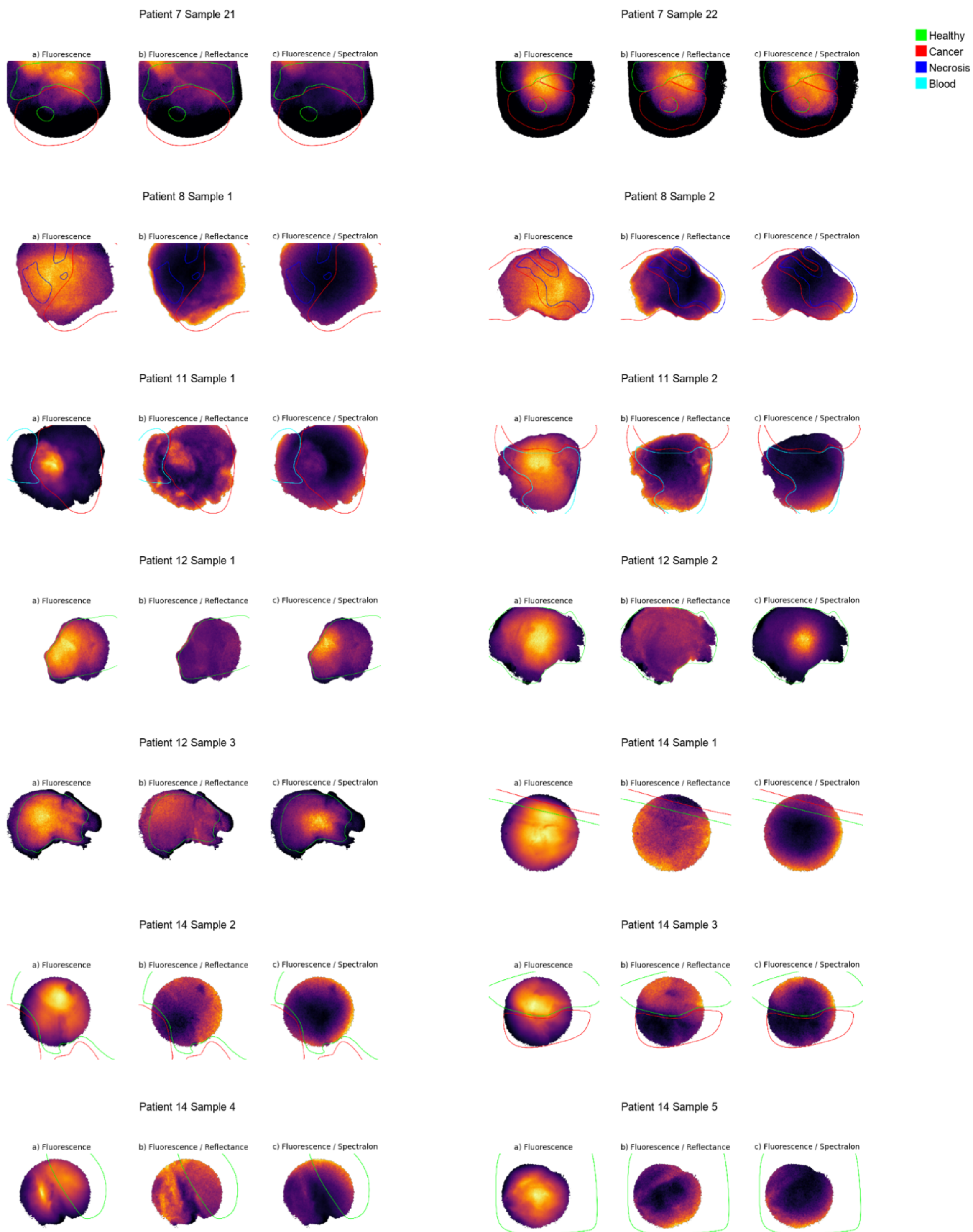

Supplementary Figure 1:

The ex vivo image panels acquired from all patients using the wide-field near-infrared fluorescence and reflectance imaging system. Panels show: (a) raw fluorescence image

acquired under 808 nm excitation through the 850 nm long-pass filter; (b) corresponding diffuse reflectance image at 808 nm; (c) reflectance image normalized by the illumination profile obtained from a Spectralon standard, illustrating spatial correction for non-uniform excitation.
